# Run length encoding based wavelet features for COVID-19 detection in X-rays

**DOI:** 10.1259/bjro.20200028

**Published:** 2021-02-02

**Authors:** Ahmad Sarhan

**Affiliations:** 1 Department of Computer Engineering, Amman Arab University, Amman, Jordan

## Abstract

**Objectives:**

Introduced in his paper is a novel approach for the recognition of COVID-19 cases in chest X-rays.

**Methods:**

The discrete Wavelet transform (DWT) is employed in the proposed system to obtain highly discriminative features from the input chest X-ray image. The selected features are then classified by a support vector machine (SVM) classifier as either normal or COVID-19 cases. The DWT is well-known for its energy compression power. The proposed system uses the DWT to decompose the chest X-ray image into a group of approximation coefficients that contain a small number of high-energy (high-magnitude) coefficients. The proposed system introduces a novel coefficient selection scheme that employs hard thresholding combined with run-length encoding to extract only high-magnitude Wavelet approximation coefficients. These coefficients are utilized as features symbolizing the chest X-ray input image. After applying zero-padding to unify their lengths, the feature vectors are introduced to a SVM which classifies them as either normal or COVID-19 cases.

**Results:**

The proposed system yields promising results in terms of classification accuracy, which justifies further work in this direction.

**Conclusion:**

The DWT can produce a few features that are highly discriminative. By reducing the dimensionality of the feature space, the proposed system is able to reduce the number of required training images and diminish the space and time complexities of the system.

**Advances in knowledge:**

Exploiting and reshaping the approximation coefficients can produce discriminative features representing the input image.

## Introduction

In late 2019, the coronavirus disease (COVID-19) was discovered in China. The World Health Organization (WHO) declared COVID-19 a global pandemic in March 2020.^1^ As of October 2020, the reported number of COVID-19 cases surpassed 40 million, leaving more than 1.1 million deaths worldwide.^2^

This paper introduces a new method for the detection of COVID-19 cases employing support vector machines (SVMs) and the discrete Wavelet transform (DWT). The DWT is well-known for its energy compression power.^3^ A SVM is a commonly used machine learning (ML) program that has been extensively employed in classification tasks.^4^ To extract only high-energy Wavelet approximation coefficients, the proposed system introduces a new coefficient selection method that employs thresholding, run-length encoding (RLE), and zero-padding. The generated code vector is employed as the feature vector symbolizing the input chest X-ray (CXR) image. It is demonstrated through experiments that exploiting and reshaping the Wavelet approximation coefficients, can produce highly discriminative features symbolizing the input image.

Using the DWT, the proposed system decomposes the chest X-ray image into a set of approximation coefficients that contain a few high-energy coefficients. The proposed system extracts these high-energy coefficients through a new coefficient selection technique that employs hard-thresholding with a non-negative threshold. The thresholded vector contains the wanted coefficients with many undesired zeros. The proposed coefficient selection method eliminates the unwanted zeros in the thresholded vector by employing the RLE scheme. The generated code vector is employed as the feature vector symbolizing the input chest X-ray image. After applying zero-padding to unify their lengths, the feature vectors are passed to a SVM for classification (COVID-19 or normal).

Also presented in this paper are the advantages and disadvantages of common testing techniques for COVID-19. The discussed methods include genomic sequencing, antibody tests, and nucleic acid tests. This paper also presents a comparison review of accuracies and dataset sizes for different image-based methods proposed for diagnosing COVID-19.

Among RNA viruses, the genome of the coronavirus is considered the longest known genome.^5^ The most common symptom of COVID-19 is fever. Other symptoms may include loss of smell, shortness of breath, cough, and fatigue. Pneumonia and acute respiratory distress syndrome are common complications.^6^ The period from COVID-19 exposure to the occurrence of symptoms is typically about 5 days but may vary from 2 to 14 days.^7^ Asymptomatic people, or people who do not show symptoms, can also spread the virus. Specifically, the first 3 days after the symptoms onset, the virus is most contagious.^8^

The virus spreads mostly to people gathered in the same area. Small respiratory particles (droplets) developed primarily by sneezing, talking, and coughing, can cause the spread of the COVID-19 virus. There is no evidence that the droplets travel through air over long distances. They rather come down to the ground or fall onto surfaces.^9^ Touching the face after touching a contaminated object or surface may cause a person to get infected by COVID-19.^10,11^

A measure of how easily a disease spreads, is given by the reproductive number R0, pronounced “R-naught”. The reproductive number of COVID-19 is in the range between 2 and 3.^12^ The number is not fixed and may vary with time. A lower R0 implies that fewer people will be infected during the outbreak. A higher number means more people will be infected over time. The following examples illustrate the meaning of R0:

- If R0 is bigger than 1, each sick person will infect, on average, more than one person. As a result, the outbreak will continue to grow and lockdown measures should not be alleviated.
- If R0 is 1, each infected person infects, on average, just one more person. Over time, the number of infected people will not vary.
- If R0 is less than 1, each infected person will infect, on average, fewer than one person. Therefore, the number of infected individuals will decrease over time. In this case, lockdown measures may be alleviated.

Some countries like Sweden resorted to the herd immunity approach to seek protection from COVID-19. Herd immunity is achieved when a large part of a community become immune to a specific infectious disease.^13,14^ Consequently, the disease has nowhere to go and stops spreading. Two scenarios may lead to herd immunity:

1. Many people get infected with the disease. Those who do not die from the disease, develop natural immunity to it. When the body is exposed to a virus or bacteria, it normally makes antibodies to fight the infection. When the body recovers, it keeps these antibodies, which will be used to defend against another infection.
2. Many people are vaccinated against the disease in order to build immunity against it. Vaccines make the body thinks that a bacteria or virus has attacked it; and therefore, the immune system develops antibodies. When the body encounters that virus or bacteria again, it can defeat it.

The spread of COVID-19 is normally controlled by quarantine in combination with other control strategies.^15^ The aim of quarantine is to confine people who might have been infected by the virus. People in quarantine should stay home, keep themselves away from others, and follow guidance from their local health authorities. A major benefit of quarantine is to prevent spread of the virus that may occur from symptomatically and asymptomatically infected individuals.^16^ Many countries require passengers to quarantine themselves for 14 days. The recommended 14-day rule is a commonly accepted practice by most countries in the world.^17^

Just as with other infectious diseases, correct sample accumulation is a very important step in the laboratory detection of COVID-19. Appropriate samples include lower respiratory tract samples, upper respiratory tract samples, whole blood samples, serum, and stool samples. The most commonly used samples are the respiratory secretions.^18,19^

As of October 2020, there are no antiviral treatment, vaccines, or recommended medicines for COVID-19. A rapid diagnosis of COVID-19 with high accuracy is still unavailable.^20^ The currently available COVID-19 diagnostic tests are broadly based on the following six different approaches:

1. RT-PCR (reverse transcription polymerase chain reaction): a viral test based on reverse transcription of RNA into DNA. It immediately tests for the presence of the virus RNA. RT-PCR test detects the presence of the virus itself.^21^ RT-PCR is considered the standard method for detecting COVID-19.^22^
2. LAMP (loop-mediated isothermal amplification): a gene amplification technique that discovers whether or not viral RNA is present in the patient’s samples.^23^
3. Lateral flow (antibody tests): handheld single-use assays providing very fast results for an individual patient (in less than 15 min).^24^ Antibody (serology) tests checks if a person had COVID-19 in the past by looking for antibodies, which are normally produced in response to infection.^25^
4. Enzyme-linked immunosorbent assay (ELISA): simple and quick assays that are easily read.^26^ ELISAs use enzymes linked to antibodies that can adhere to the tested molecule and cause a change in color, which can subsequently be measured by a device.
5. Genomic sequencing: investigates the genetic information found within the DNA or RNA of a virus. It enables scientists to compare the virus sample taken from a patient, with other people.^27,28^
6. Chest imaging: new reports from China indicate that chest images produced by CT, X-ray, and ultrasound can help diagnose the COVID-19 disease.^29–32^ Chest imaging is not normally used as a first choice tool to diagnose or screen for COVID-19. The advantages and disadvantages of the COVID-19 tests are summarized in Table 1.

**Table 1.** Advantages and disadvantages of COVID-19 diagnosis tests

Approach	Advantages	Disadvantages
RT-PCR	Sensitive, reliable, and fast.	Expensive, complex, efficiency depends on the adequate amount of the viral RNA sample.
LAMP	Simple, reliable and sensitive if the samples are acquired while an infection is going on.	· LAMP tests determine if an active virus is present; and therefore, cannot find out if a person had the disease. · New technology which does not have much background behind it.
Antibody tests	· Simple and rapid. · Can check the immunity of people to the virus by revealing if they have already encountered the virus without their own knowledge.	· Cannot determine if the person is currently infected with COVID-19 as infected persons do not have antibodies. · These tests can yield a false negative result if the test is conducted before the development of antibodies. · The antibody test can generate a false positive result if antibodies to other coronaviruses are present.
ELISA	· More specific than Lateral flow tests. · Simpler than other procedures and uses less expensive equipment. · Allows collection of samples from different spots in the body (not restricted to nasal swabs).	· Time consuming. · Low sensitivity.
Chest Imaging	· Can utilize ML algorithms, which are available for imaging applications, for accurate and automatic detection of COVID-19. · Can provide radiologists with visual information related to the viral infection. · Chest imaging systems are widely available.	· Cannot correctly discriminate between COVID-19 and other respiratory infections, such as Influenza. · Can generate false negative results since a substantial portion of COVID-19 patients have normal chest x-rays or CT scans; and therefore, their imaging result could falsely indicate that they are healthy. · Since COVID-19 is very contagious, the use of imaging equipment by COVID-19 patients, may cause a health hazard.
Genomic sequencing	· Very sensitive and specific. · Provide detailed information.	· Require high expertise. · Involve sophisticated Lab.

LAMP, loop-mediated isothermal amplification; RT-PCR, reverse transcription polymerase chain reaction.

## Machine learning techniques for image based detection of COVID-19

Most of the automatic COVID-19 diagnosis techniques that are based on chest imaging, employed artificial intelligence (AI) and ML methods. These systems are also known as computer-aided diagnosis (CAD) methods.

Ozturk et al^33^ worked on COVID-19 detection in X-rays by employing the You only look once (YOLO) convolutional neural network (CNN). YOLO, used for target detection, is a real-time network configured based on the Darknet model. They reported an 87% classification accuracy for multiclass cases. For the two-class cases (normal or COVID-19), they achieved an accuracy of 98%.

Barstugan et al^34^ worked on the detection of COVID-19 in CT images. Using a data set composed of 150 CT scans, they generated four different data sets by taking patches whose sizes are 16 × 16, 32 × 32, 48 × 48, and 64 × 64. For feature extraction, they used the following techniques: Grey-Level Size Zone Matrix (GLSZM), DWT, Grey Level Co-occurrence Matrix (GLCM), Local Directional Pattern (LDP), and Grey Level Run Length Matrix (GLRLM). SVM was used to classify the obtained features. A 10-fold cross-validation was employed and produced a maximum detection accuracy of 99%.

Wang et al^35^ proposed the COVID-Net, a deep learning method to detect COVID-19. They used 13,870 COVID-19 patients to build a very large database that consisted of 13,975 chest X-ray images. They reported a 93% detection accuracy and a sensitivity of 91%. They compared the performance of COVID-Net to the performances of the following deep neural networks: ResNet (Residual Network)−50 and VGG-19.

Hemdan et al^36^ worked on the classification of COVID-19 cases in X-rays by proposing a COVIDX-Net model, which is composed of the following seven CNN architectures: Xception, VGG19, InceptionV3, InceptionResNetV2, ResNetV2, DenseNet201, and MobileNetV2. Their experiments were validated on a small data set that contained 50 chest X-ray images (25 COVID-19 cases and 25 normal cases). They used 80% of the images for training and 20% for testing.

Sethy et al^37^ employed SVM combined with the ResNet50 model to detect COVID-19 cases in chest X-rays. Their data set consisted of 25 normal images and 25 COVID-19 images. The images were obtained from Kaggle, GitHub, and Open-I repositories. They reported an accuracy of 95%. They also obtained a sensitivity of 97% and a specificity of 93%. Their false positive rate (FPR) was 65%. They employed a 60:20:20 testing, training, and validation ratios.

Ioannis et al^38^ used the transfer learning procedure with a deep learning model, for the automatic diagnosis of COVID-19. Their data set consisted of 1427 chest X-ray images that contained 700 pneumonia images, 504 normal images, and 224 COVID-19 images. They reported a 97% classification accuracy.

Narin et al^39^ proposed five pre-trained CNN models for the detection of COVID-19 in chest X-rays. Specifically, they suggested the following models: Inception-ResNetV2, ResNet50, InceptionV3, ResNet152, and ResNet101. Their data sets contained four classes: bacterial pneumonia, viral pneumonia, COVID-19, and normal. In some of their experiments, they used 20% of the images for testing and the remaining 80% for training. When implementing three binary classifications based on a fivefold cross-validation, they reported that the ResNet50 model produced the highest classification accuracy (99%).

We note that a proper benchmark data set for COVID-19 detection in chest X-ray/CT images, is necessary but is currently unavailable. Presented in in Table 2 is a summary of the accuracies and sizes of training and test sets, for some of the proposed methods that used X-ray scans in automatic diagnosis of COVID-19.

**Table 2.** Dataset size and accuracy of COVID-19 diagnosing models in chest X-ray images

Ref.	Accuracy	Training set	Test set
Hemdan et al.^36^	83%	40	10
Sethy et al.^37^	95%	160	54
Ioannis et al.^40^	99%	3514	391
Ioannis et al.^41^	98%	1284	143
bbas et al.^42^	95%	137	59
Zhang et al.^43^	96%	100	764
Biraja et al.^44^	90%	4753	1188
Chowdhury et al.^45^	99%	304	85
Wang et al.^46^	92%	1,6756	210
Halgurd et al.^47^	98%	263	263
Parnian et al.^48^	96%	764	100
Ezzat et al.^49^	98%	114	31
Karim et al.^50^	93%	1,1896	5099
Zahangir et al.^51^	99%	5216	45
Eduardo et al.^52^	93%	1,6546	210
Oh et al.^53^	90%	354	99
Lawrence et al.^54^	94%	410	242
Khan et al.^55^	89%	1125	126
Basu et al.^56^	95%	718	175
Li et al.^57^	97%	1791	448

## Methods and materials

**Figure 1.**
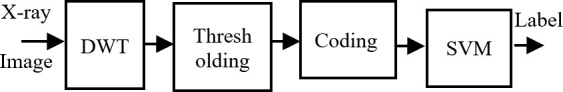
Block diagram of the proposed system. DWT, discrete Wavelettransform; SVM, support vector machine.

The proposed system employs SVM to classify the features extracted from Wavelet approximation coefficients using a new coefficient selection scheme. Figure 1 depicts a block diagram of the proposed system.

This study is based on X-ray imaging of the chest to diagnose COVID-19.

### Medical imaging modalities

The most commonly used medical imaging modalities include:

- X-ray imaging: the oldest and the most commonly used imaging technique. X-rays are high-frequency electromagnetic radiations that can pass through the body. The X-rays that penetrate through an object are collected behind the object by digital sensors or a photographic film. The X-ray method is typically employed in diagnosing the skeletal system.
- CT: uses a computer to combine a set of X-ray images taken at various angles around the body, to form slices (cross-sectional images).
- Ultrasound imaging: reflected sound waves are used by the computer to generate images of body organs and other body parts.
- MRI: high-intensity magnetic fields and radiofrequencies are used by the computer to generate images of the body’s internal parts.

#### The pros and cons of imaging modalities

The advantages and disadvantages of imaging modalities are summarized in Table 3.

**Table 3.** Advantages and disadvantages of imaging modalities

Approach	Advantages	Disadvantages
X-ray	Cheap and easy to use.	· Not very safe because ionizing radiation can cause cancer or lead to cell mutations. · Does not generate detailed images. · Used to image bones only.
CT	Very detailed and precise.	More ionizing radiation than X-ray.
Ultrasound	Safe, affordable, and simple.	· Low image resolution. · Increase tissue temperature.
MRI	· Can image any body part. · Safe- no ionizing radiation.	· Expensive. · Uncomfortable for patients.

#### Digital radiography *vs* computed radiography

Digital radiographic images can be obtained using digital radiography (DR) and computed radiography (CR) technologies. Both DR and CR techniques use digital systems to produce a digital image. DR uses flat panel transducers (sensors) that convert X-ray intensities to proportional voltages. A microcontroller or a computer processes these data (voltages) to produce a digital image.

CR, on the other hand, employs a photostimulable-phosphor (PSP) plate. When an imaging plate is exposed to X-rays, the energy of the incoming radiation is absorbed in a special phosphor layer to form a latent image. A scanner is then employed to capture the latent image from the storage plate by first stimulating the plate with a fine laser beam. When stimulated, the plate releases the stored energy by emitting blue light whose intensity is proportional to the amount of radiation absorbed in the exposure phase. The light is then detected by a photomultiplier (PMT) which is an optical transducer that coverts light to analog voltage. Using an analog-to-digital converter (ADC) IC (integrated circuit) chip, the PMT output is converted to a digital image.

Once the latent image is read, the imaging plate can be erased and reused. Erasure of the latent image can be achieved by subjecting the plate to a high-intensity light. Normally, the plate can be used for about 100 times.

#### X-ray artifacts

Radiography systems may produce several artifacts. An artifact refers to an appearance or something seen on a radiograph that is not really present but appears due to a fault that occurred somewhere in the imaging chain. The fault may be caused by equipment defect, the operator of the imaging equipment, or by a peculiarity of the modality itself. The fault may also be caused by external things such as monitor wires and patient clothing. Image noise is the most common artifact and is intrinsic to every modality. Although image noise can be attenuated, it cannot be completely removed. The common computed/digital radiography artefacts^58^ are listed in Table 4.

**Table 4.** Typical radiographic artifacts

Signal processing	· Ghosting or image lag-DR. · Presence of previous latent image due to incomplete erasure-CR. · Detector saturation thresholds - DR/CR.
Detection	· Damage of Imaging plate-CR. · Dust or dirt in reader-CR · Dead pixels/lines- DR/CR
Acquisition	· Radiation scattering through back of the detector -DR/CR. · Beam hardening- DR/CR. · Grid interference patterns-DR/CR. · Under/overexposure- DR/CR ·
Image Transmission	· Readout disruption-CR/DR. · Image stitching- CR/DR
Mains electricity	Mains power cables produce 50/60 Hz signal (radiation). This electromagnetic interference (EMI) can be absorbed by the measurement leads to form noise in the measurement. The 50/60 Hz signal has narrow frequency bands; usually outside the band of the desired signal, and can be normally removed using a band-pass filter.
Instrumentation	Thermal noise originates from the electronic circuit itself.
Experimental error	Generated by undesired /uncontrolled change in the setup of the experiment.

EMI, electro magnetic interference.

CT artifacts can be reduced by proper planning and procedure, but usually cannot be completely. wiped out.^59^ CT artifact removal in ML algorithms for CT imaging, has not been adequately addressed. Most of the research in this field has mainly focused on grid artifacts and artifacts caused by metalware. Metal artifact reduction (MAR) algorithms are used to enhance the quality of CT images in patients with metal implants.^60^ Grid artifacts are removed in the frequency domain by filtering since grid artifacts occupy a narrow range of frequencies.^61^

Wang et al^46^ performed an audit on COVID-Net to validate that its classification was not based on imaging artifacts and embedded markup symbols. Karim et al suggested to eliminate textual artefacts from chest X-ray images using thresholding to remove very bright pixels. They also employed image standardization and normalization.^50^ Pixel normalization refers to scaling pixel values to the range 0–1. Pixel standardization refers to scaling pixel values to have a zero mean and unit variance. These processes can be performed either per data set (featurewise) or per image (samplewise). In other areas of biomedical signal processing, such as in the field of physiological signals, pre-processing methods and adaptive filtering are currently the main techniques used in removing physiological signal artifacts.

In the proposed system, the training process adaptively learns to remove the common imaging artifacts. The relatively high accuracy produced by the proposed system demonstrates its ability to detect artifacts related to digital radiography.

**Figure 2.**
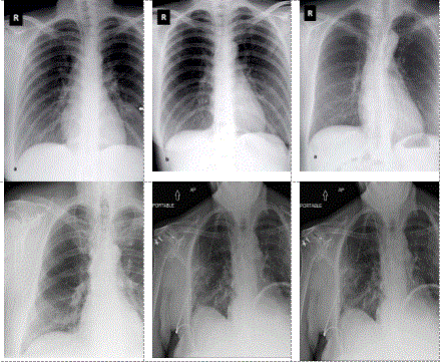
Samples from our data set: (Top): COVID-19 cases and (Bottom): normal cases.

At the time of conducting this study, only a few X-ray and CT scans were publically available. The chest X-ray images symbolizing COVID-19 cases were gathered from Cohen.^62^ Cohen collected the COVID-19 chest X-ray images from various sources. Cohen’s database is composed of 125 COVID-19 chest X-ray images. The images had different formats (jpeg, jpg, and png). 88 images were gathered from Cohen database. Figure 2 (top) depicts sample COVID-19 images that were taken from Cohen database.

Cohen database, however, does not comprise normal (negative) cases. In this study, the normal chest X-ray images were acquired from the Chest X-ray8 database offered by Wang et al.^63^ Chest X-ray8 included more than a thousand frontal view X-ray images. For this study, only 88 normal (no-finding) images were drawn from this database. Figure 2 (bottom) shows sample images collected from Chest X-ray8 database. Therefore, our data set consisted of 176 chest X-ray images (88 normal images and 88 COVID-19 images). Other public COVID-19 chest X-ray images can be found in.^64–66^

It is important to note that in the ideal scenario, all images should be taken using the same equipment and under the same conditions. However, in this COVID-19 study, chest X-ray images were very restricted; at least when this report was first prepared. Theoretically, image classification is independent of the imaging modality and is affected by the input image. However, the image quality (contrast resolution, spatial resolution, artifacts, and noise) is greatly dependent on the imaging modality.

Originally, the images making our data set were of different grayscale and spatial resolutions. Before further processing, all images were first changed to 8-bit intensity images with a 512 × 512 spatial resolution. Different image sizes and intensity resolutions will have different statistical properties; and therefore when analyzed using the proposed system, will not produce the same optimum parameters (decomposition level and threshold values) reported in this paper. The first operation of the proposed system is to decompose the input chest X-ray image using the DWT.

### Discrete Wavelet transform and feature extraction

**Figure 3.**
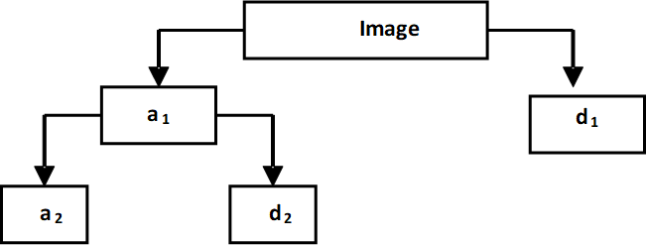
Wavelet decomposition tree: a_1_ and d_1_ represent the approximation and detail coefficients at level 1, respectively.

The DWT or Wavelet decomposition, is a mathematical function (mapping) that generates another representation of the input signal or image.^67,68^ The DWT is well-known for its energy compression power. The Wavelet decomposition tree, depicted in Figure 3, shows the main functions executed by the DWT acting on an input image. The input image, at the first level of decomposition, dissolves into both approximation and detail coefficients. While the low frequency contents of the input image are carried by the approximation coefficients, the detail coefficients hold the high-frequency information. At the second level of decomposition, the approximation coefficients develop two groups of approximation and detail coefficients, whose lengths are equal to half of the length of the original approximation vector. This procedure continues to break the approximation coefficients into two new vectors for each subsequent level of decomposition.^69–71^

Unlike Fourier and other signal transforms, the DWT exhibits a great energy condensation property as most of the energy of the transformed image is deposited in few ultra large approximation coefficients. This characteristic indicates that small coefficients can be set to zeros without establishing a huge distortion in the inverse-transformed image. The energy compactness attribute of DWT has been successfully utilized in image compression schemes, such as the jpeg compression scheme.^72^ In data compression applications, only Wavelet coefficients which contain most of the signal energy are retained for use in the signal reconstruction.

In the proposed system, we exploit this energy compression property of the DWT to form a discriminative feature vector representing the input image. High-energy coefficients are extracted in the proposed system using the hard-thresholding scheme, given by Equation 1:

(1) $$C(i)=\left\{ \begin{array}{ll} 0 & if\;|C(i|§lt;T\\ C(i & otherwise) \end{array}\right.$$

where, $\hat{C}$ (*i*), and *C*(*i*) are the *i*th approximation coefficient after and before thresholding, respectively; and *T* is the threshold value.

Equation 1 indicates that the elimination of small-valued coefficients can be achieved by setting to zeros all coefficients whose values are less than a certain threshold value. An illustration of the hard-thresholding technique, using a threshold value of 3.1, shown in Table 5..

**Table 5.** Hard-thresholding scheme

Input vector	Output vector
−11	−11
−2.9	0
3.2	3.2
2.3	0
300	300

The input and output vectors represent the Wavelet approximation coefficients before and after thresholding, respectively. By selecting a non-negative threshold, the small approximation coefficients can be reset to zeros. To determine the optimum threshold value, we suggest inspecting the histogram of the approximation coefficients or using statistical moments of the approximation coefficients. Other thresholding schemes are discussed in Wei and Burrus,^73^ Chang and Vetterli,^74^ Donoho,^75^ Poornachandra and Kumaravel.^76^

The standard level-thresholding mechanism produces a lot of zeros, generating a vector that is too large to carry a few discriminative features. The zeros are eliminated in the proposed coefficient selection technique by utilizing the RLE scheme.

### Run-length encoding

**Figure 4.**
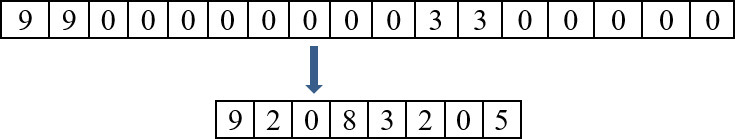
RLE scheme. (top): input vector and (bottom): output vector. RLE, run-length encodingThe RLE generated vector is employed as the feature vector representing the input image.

The RLE scheme, patented by Hitachi, is employed in JPEG, MPEG, H.261, and H.263 compression methods.^77^ The RLE methods were first used in 1967 in the analog signal transmission for television applications. Basically, RLE replaces a string of identical values by codes to indicate the value and the number of times it occurs. To illustrate the RLE scheme employed in this study, consider an approximation vector consisting of 50 zeros. RLE converts it to two numbers. The first number is 0, which indicates the string zeros and the other number is 50, which indicates the number of zeros. Figure 4 depicts an illustration of the RLE scheme used in this study.

After applying zero-padding to unify their lengths, the feature vectors are applied to a SVM for classification (normal or COVID-19).

### Support vector machines

**Figure 5.**
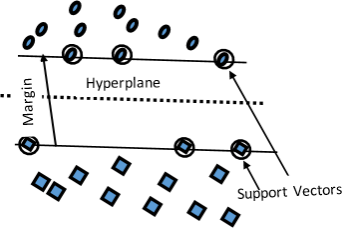
Support vector machine for a 2D data. 2D, two-dimensional.

A SVM is considered one of the most commonly used AI algorithms. SVMs are ML methods that were first introduced by Cortes and Vapnik.^78^ Regression and classification are the most common applications of SVMs. A SVM classifies data by first detecting the closest vectors (support vectors) among the data comprising the classes. Using supervised learning, the SVM classifier then determines the optimum hyperplane that isolates the data points of the classes by producing the widest possible margin (Figure 5). In two-dimensional (2D) data, the hyperplane reduces to a simple line.

SVMs can manage linear and non-linear tasks. Linear problems are problems where data can be easily separated by drawing a hyperplane or straight line. In non-linear problems, data cannot be easily separated with a linear line.

SVMs were originally designed to be binary or two-class classifiers. However, SVMs have been altered to tackle data composed of more than two classes. A SVM kernel is a function used in SVM to facilitate problem solving. Kernels provide shortcuts (tricks) to go around complicated computations. A kernel allows for mapping the problem to higher dimensions in order to perform simpler computations. An infinite number of dimensions can be obtained using kernels. The performance of SVM can be altered by choosing a different kernel function.

The Gaussian kernel, given by Equation 2, was implemented by the SVM in the proposed system.

(2) $$k(x,y)=exp-\frac{{\left\|x-y\right\|}^{2}}{2\sigma^{2}}$$

where σ is a user-defined variance.

The Gaussian kernel is a general-purpose kernel. It can be used when there is no prior information about the data. Other kernels include the Polynomial kernel, Gaussian radial basis function (RBF), Laplace RBF kernel, Hyperbolic tangent kernel, and Sigmoid kernel.

## Discussion and results

To avoid overfitting, fourfold cross-validation was employed in the assessment of the system’s accuracy. Hence, our data set was divided into four non-overlapping folds. Four experiments were performed. Each of the four folds was used in one of the experiments as a test set. The remaining three folds were used as a training set. The accuracy of each experiment was calculated and the average of the four accuracies was reported as the system’s overall accuracy.

**Figure 6.**
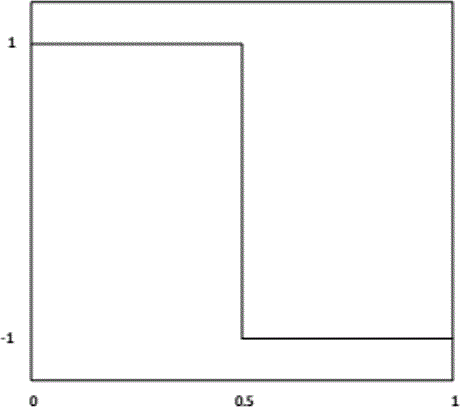
The Haar (db1) Wavelet.

In the first investigation, the accuracy is computed *vs* the Wavelet decomposition level. This experiment used the Haar Wavelet. The Haar Wavelet, also known as the Daubechies 1 (db1) Wavelet, is considered the simplest Wavelet. The Haar Wavelet is depicted in Figure 6.

**Figure 7.**
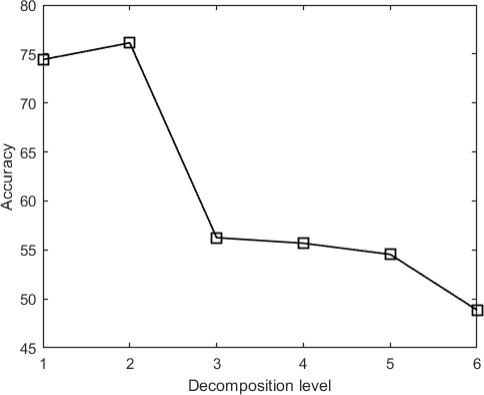
Accuracy *vs* Wavelet Decomposition Level

Figure 7 depicts the accuracy of the proposed system *vs* decomposition level. Figure 7 shows that a maximum accuracy of 76% is achieved for a decomposition level of 2. Accuracy is defined in this experiment as the rate of correct detections.

**Figure 8.**
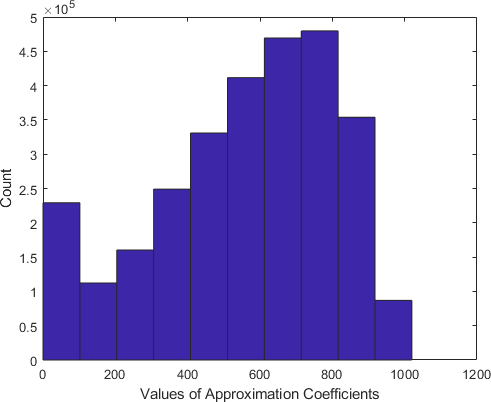
Histogram of the approximation coefficients at level-2.

Since the optimum decomposition level is 2, we further investigate the approximation coefficients at level 2. Figure 8 shows the histogram of the approximation coefficients of all the 176 images comprising the employed data set, decomposed at Level 2 using the Haar Wavelet. Figure 8 indicates that only few approximation coefficients have high magnitudes. Specifically, a small subset of the approximation coefficients has magnitudes greater than 900.

**Figure 9.**
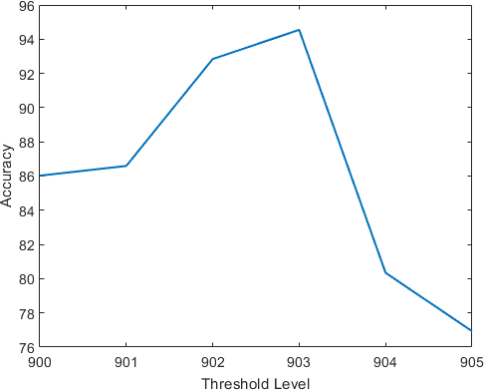
Accuracy *vs* threshold value, using the Haar Wavelet and a decomposition level of 2.

Next, we investigate the accuracy using threshold values around 900. Specifically, in the experiment of Figure 9, threshold values were investigated in the range between 900 and 905, using a decomposition level of 2 and the Haar Wavelet. In the experiment, approximation coefficients whose absolute values are less than the variable threshold value, were set to zeros.

The resultant thresholded vector, composed of mostly zeros, is then encoded using the RLE scheme, to produce a code vector. The code vector is used as the feature vector symbolizing the input chest X-ray image. After applying zero-padding to unify their lengths, the feature vectors are passed to a SVM for classification (normal or COVID-19). Figure 9 depicts the accuracy as a function of threshold value. Figure 9 shows that the threshold value of 903 produces the maximum accuracy of 94%.

Specificity (SP), sensitivity (SE), and accuracy (AC) are used here to assess the performance of the proposed system.

Accuracy, given by Equation 3, is defined as the ratio of non-occurrences that are accurately rejected and the real occurrences that are accurately identified, among all occurrences and non-occurrences.

(3) $$AC=\frac{(TP+TN)\times 100}{\left(TP+FN+FP\right)}$$

Sensitivity (also called true positive rate) is the fraction of positive occurrences that are accurately discovered by the system:

(4) $$SE=\frac{TP\times 100}{FN+TP}$$

Specificity (true negative fraction) measures the power of the system to accurately discover those who do not have the disease.

(5) $$SP=\frac{TN\times 100}{FN+TP}$$

where,

TP: True positive samples (predicts COVID-19 as COVID-19).

FP: False positive samples (predicts normal as COVID-19).

TN: True negative samples (predicts normal as normal).

FN: False negative samples (predicts COVID-19 as normal).

The prevalence (PR) is calculated using the following equation:

(6) $$PR=\frac{AC-SP}{SE-SP}$$

Table 6 shows the calculated PR, SE, SP, and AC.

**Table 6.** Performance measures of the proposed system

Number of cases	AC	SP	SE	PR
100	94%	90%	92%	2

AC, accuracy; PR, prevalence; SE, sensitivity;SP, specificity.

Table 6 illustrates that the proposed system delivers high specificity and sensitivity ratios. The presence of other diseases in the COVID-19 chest image is a challenge to any classifier. The success of the classifier depends greatly on the training data. In general, the greater the number of lung images in the dataset, that are correctly classified with the corresponding diseases, the higher the accuracy of the classifier.

It is important to point out that the CNN classifier, the main competitor to the proposed system, cannot challenge the proposed system in the COVID-19 detection application. The majority of the proposed CNN systems feed the whole input image to the CNN classifier. In this study, image size is 512 × 512, and when the whole image is fed as input (no feature extraction), the size of the feature space dimension is

512 × 512=262,144.

**Figure 10.**
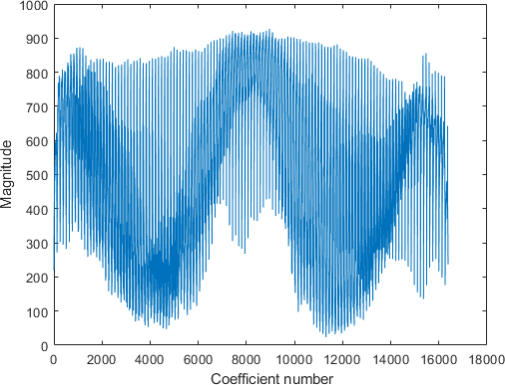
Approximation coefficients representing a sample COVID-19 image in the data set.

The feature space dimension for the proposed system, on the other hand, is the length of the approximation vectors, which is 16,384 coefficients, as indicated by Figure 10. Figure 10 depicts the approximation coefficients representing a sample COVID-19 chest X-ray image used in our dataset, decomposed at level 2 using the Haar Wavelet.

Figure 10 indicates that the length of the approximation vector is 16,384 coefficients. We hypothesize that for an n x m image decomposed at level L, the length of the approximation coefficient vector l is given by

(7) $$l=\frac{m\;x\;n}{2^{2L}}$$

For example, the size of the images in our dataset is 512 × 512. When decomposed at level 2, equation 7 gives 1,6384 as the size of the approximation vector, as shown below:

512 × 512/2^4^ = 1,6384.

**Figure 11.**
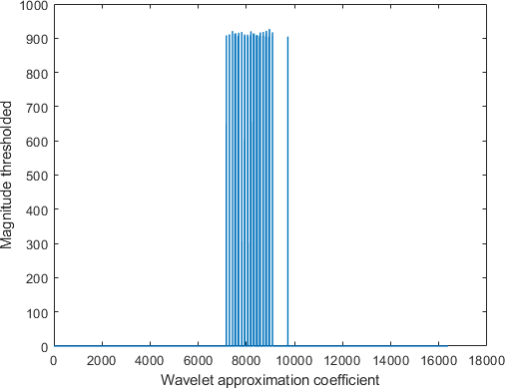
Approximation coefficients of Figure 10, thresholded using a threshold value of 904.

Figure 11 shows the approximation coefficients of Figure 10, thresholded using a threshold value of 904. Figure 11 illustrates that thresholding a vector of 16,384 approximation coefficients, retains only 104 coefficients and sets the remaining 16,280 coefficients to zeros. In other words, thresholding reduced the feature space dimension by more than 99%.

As a commonly used rule of thumb, data set size should be about 10 times its dimension.^79^ Using the 10x rule, the number of training images needed by the proposed system is

16,384 × 10=163,840.

Whereas the number of training images needed by a CNN classifier is

262,144 × 10=2,621,440, which is impractical considering the small number of COVID-19 chest images that are currently available. Typically, deep learning demands millions of training images.

Just like other CAD systems, the proposed system processes digital images to help radiologists and other medical professionals in examining medical images such CT, X-ray, ultrasound, and MRI scans. Furthermore, due to human-related factors such as tiredness, fatigue, and enormous workload, the subjective interpretation by the observer of medical images, can be delusive or insufficient. In such situations, CAD systems can be very assistive.

## Conclusion

In this paper, a novel approach to the detection of COVID-19 cases employing SVM and DWT is proposed. The DWT is appreciated for its energy compression power. To find discriminative features in the X-ray image, the proposed system utilizes DWT to decompose the input chest X-ray image into a set of approximation coefficients that include a small number of high-energy (high-magnitude) coefficients. The proposed system introduces a new coefficient selection technique that uses thresholding, RLE, and zero-padding to extract only high-energy Wavelet approximation coefficients. These features are subsequently introduced to a SVM classifier for detecting whether the input image represents a normal or COVID-19 case.

By drawing out a restricted set of discriminative features, the proposed system proves its ability to reduce the feature space dimension, which naturally leads to the minimization of required training data set size and to the reduction of space and time complexities of the system.

The performed experiments show that exploiting and reshaping the Wavelet approximation coefficients can produce discriminative features symbolizing the input image. Experiments on the used data sets obtained a recognition accuracy of 94% using a decomposition level of 2 and the db1 Wavelet.

The proposed system does not claim to offer a manufacturing-ready solution to the problem of COVID-19 detection in chest X-rays. The aim is to build upon the promising results achieved by the Wavelet features on the Cohen data set, anticipating that more COVID-19 X-ray images will be available in the future.

The author declares that no external funding was received for this work.
